# Spatial variation in AMF communities and soil properties correlates with leaf and root allelopathic potential in *Solidago canadensis* in six different locations

**DOI:** 10.3389/fpls.2026.1755243

**Published:** 2026-05-07

**Authors:** Huan Mei, Xueyi Huang, Zuofu Wei, Lifu Sun, Huanyi Yang, Xiaodong Zhang, Lijia Dong

**Affiliations:** 1 School of Life and Environmental Sciences, Shaoxing University, Shaoxing, China; 2 School of Life Science, Shanxi Normal University, Linfen, China

**Keywords:** allelopathy, AMF community, plant-soil interaction, secondary metabolites, soil abiotic properties

## Abstract

Allelopathy is a key driver of plant invasion. However, how arbuscular mycorrhizal fungal (AMF) communities and abiotic factors associated with plant-soil interactions (PSIs) influence plant chemistry and allelopathic potential remains poorly understood. We examined the allelopathic potential of leaf and root extracts of *Solidago canadensis* from 18 invaded quadrats across six sites using a *Raphanus sativus* bioassay. We then analyzed the associations among invasion intensity, AMF communities, soil abiotic properties, plant chemistry, and allelopathy. Results indicated significant spatial heterogeneity in leaf and root chemistry and their allelopathic strength. AMF community metrics and soil abiotic properties within the invaded communities were significantly correlated with variations in leaf and root chemistry (e.g., phenols and flavonoids), both of which were strongly linked to allelopathic potential. Leaf chemistry showed stronger allelopathic inhibition than root chemistry and was more responsive to variations in the soil environment. Our partial least squares path modeling supported a hypothesized PSI framework in which the degree of *S. canadensis* invasion was negatively associated with changes in the AMF community; together with soil abiotic factors, these were correlated with shifts in allelochemical production and allelopathic potential. These findings outline a correlative network through which invasive plants may modify soil biota and chemistry, a process potentially linked to their ecological dominance.

## Introduction

As globalization accelerates, plant invasion seriously threatens local biodiversity and ecosystem stability (Gioria et al., 2023). During the process of plant invasion, allelopathy and plant-soil feedbacks represent two important and increasingly interconnected processes (Callaway et al., 2004; Hierro and Callaway, 2021; Sheng et al., 2022; Lustenhouwer et al., 2024). These interactions may thereby shape competitive outcomes, population dynamics, and community structure (Bever, 2003; Suding et al., 2013). As plants both shape and respond to soil conditions, invasive species are often associated with distinct soil microbial communities and abiotic properties (Bennett and Klironomos, 2018; 2019). However, it remains unclear whether such variation in plant-soil contexts is associated with corresponding variation in the biochemical traits of invasive plants, particularly their allelopathic potential.

Plant chemistry plays a central role in plant–plant interactions through allelopathy. Allelopathy is defined as the effects, either inhibitory or stimulatory, that a plant exerts on neighboring plants or associated soil organisms via the release of allelochemicals (a subset of secondary metabolites) into the soil (Meiners et al., 2017). In the context of plant invasion, allelopathic effects are often negative, contributing to the suppression of native species. Given that the soil environment comprises diverse microbial communities and abiotic properties, the production and release of plant allelochemicals are closely linked to local environmental context, with potential implications for chemical interactions between plants (Guerrieri and Rasmann, 2024; Delory et al., 2024). Experimental studies have shown that soil microorganisms are associated with shifts in plant secondary metabolite profiles, including terpenoids, phenols, and alkaloids (Zubek et al., 2010; Singh et al., 2022; Lv et al., 2024). Likewise, soil abiotic properties such as pH, water content, organic matter, nutrient availability, and micronutrient conditions are also closely associated with plant secondary metabolism (Heimler et al., 2017; Xie et al., 2018; Liu et al., 2022; Hu et al., 2023). Together, these findings suggest that allelopathic potential should not be viewed as a fixed attribute of invasive plants, but rather as a context-dependent property that may vary across habitats and soil conditions. Although most studies have demonstrated that allelopathy can contribute to the success of alien plants, the observed variability in allelopathic strength is high, which has been partially attributed to the context-dependent nature of allelochemical production (Meiners et al., 2017; Zhang et al., 2021). However, the extent to which spatial variation in soil biotic and abiotic factors is associated with allelopathic potential in invasive plants remains largely unexplored under natural field conditions.

The importance of arbuscular mycorrhizal fungi (AMF) in modulating plant invasion success has drawn significant attention (Shah et al., 2009; Traveset and Richardson, 2014). Although the mechanisms underlying plant-AMF interactions are often linked to enhanced nutrient uptake and resistance to abiotic stress, pathogens, and herbivores (Cipollini et al., 2012; Majewska et al., 2017), their potential association with invasive plant allelochemicals production has not been well evaluated under field conditions. AMF colonization can also modulate plant defense- and signaling-related pathways, particularly jasmonic- acid- and salicylic- acid-related responses, which are frequently linked to changes in secondary metabolite production (Pozo and Azcón-Aguilar, 2007; Kaur and Suseela, 2020). Previous studies suggest that AMF can influence the accumulation of allelochemicals (e.g., phenols and flavonoids) in host plants, which may in turn affect allelopathic potential (Xie et al., 2018; Sarmiento Lopez et al., 2021; Shan et al., 2023). Additionally, AMF symbiosis enhances the transformation of nutrients such as organic N and organic P, inducing an increase in plant secondary metabolites (Tao et al., 2019; Singh et al., 2022). Thus, AMF may represent an important biotic component associated with spatial heterogeneity in invaders’ allelopathic potential under natural soil contexts. Considering that invasive plant–soil interaction (PSI) effects are greatly co-affected by nutrients, mutualism, and allelopathy in abiotic and biotic contexts (Bennett and Klironomos, 2019), we predicted that AMF community and soil abiotic factors, including nutrients, would covary with changes in the strength of an invasive plant’s allelopathy.

*Solidago canadensis* L. (Asteraceae), native to North America, is a perennial herb forming large clonal colonies that may reduce the abundance of native vegetation (Abhilasha et al., 2008; Dong et al., 2021). It has been reported to exert strong allelopathic effects on neighboring native plants, but the strength of allelopathy often varies across different studies (Abhilasha et al., 2008; Kato-Noguchi and Kurniadie, 2022). In addition, *S. canadensis* may gain greater competitive advantages through symbiosis with AMF than native plants (Lekberg and Callaway, 2022; Xu et al., 2024). This species therefore provides an ideal model for investigating the spatial covariation between soil factors and allelopathic potential in an invasive plant.

Previous studies have largely emphasized AMF as potential targets of allelochemicals, while spatial variation in AMF communities may also be associated with shifts in allelochemical expression across environments (Barto et al., 2011; Cantor et al., 2011; Lankau, 2010; Cheng et al., 2022; Guo et al., 2023). To explore how spatial variation in AMF communities and soil abiotic conditions relates to allelopathic potential under natural invasion contexts, we surveyed six sites invaded by *S. canadensis* in China. Rhizosphere and bulk soils were collected to characterize AMF communities and soil abiotic properties, while leaf and root tissues were used to assess allelopathic potential via HPLC-based chemical profiling and standardized bioassays. Specifically, we tested three predictions: (1) leaf and root chemistry, and their corresponding allelopathic potential, exhibit significant spatial variation; (2) AMF community and/or soil abiotic factors are significantly associated with this spatial variation; and (3) leaf and root allelopathic potential differ in strength and are associated with partially distinct biotic and abiotic correlates. This study provides novel field evidence linking PSI-related factors (soil abiotic properties and AMF communities) to spatial variation in allelopathic potential.

## Materials and methods

### Study species

To evaluate the allelopathic potential of *S. canadensis* across a range of invasion environments, a standardized germination bioassay was employed following the methods of Meiners et al. (2017). This approach allows for comparing differences in allelopathy within plant species under different environmental conditions (Ladwig et al., 2012). In the bioassay, we chose the radish species (*Raphanus sativus* L., Brassicaceae, an annual herb) as the target species, which has been shown to be sensitive to allelopathy and has been widely used in many allelopathy-related studies (Butcko and Jensen, 2002; Pisula and Meiners, 2010; Meiners et al., 2017). Seeds were purchased from a commercial seed supplier (Cangzhou Hushou Agricultural Technology Co., Ltd., China).

### Field survey and sampling

**Figure 1:**
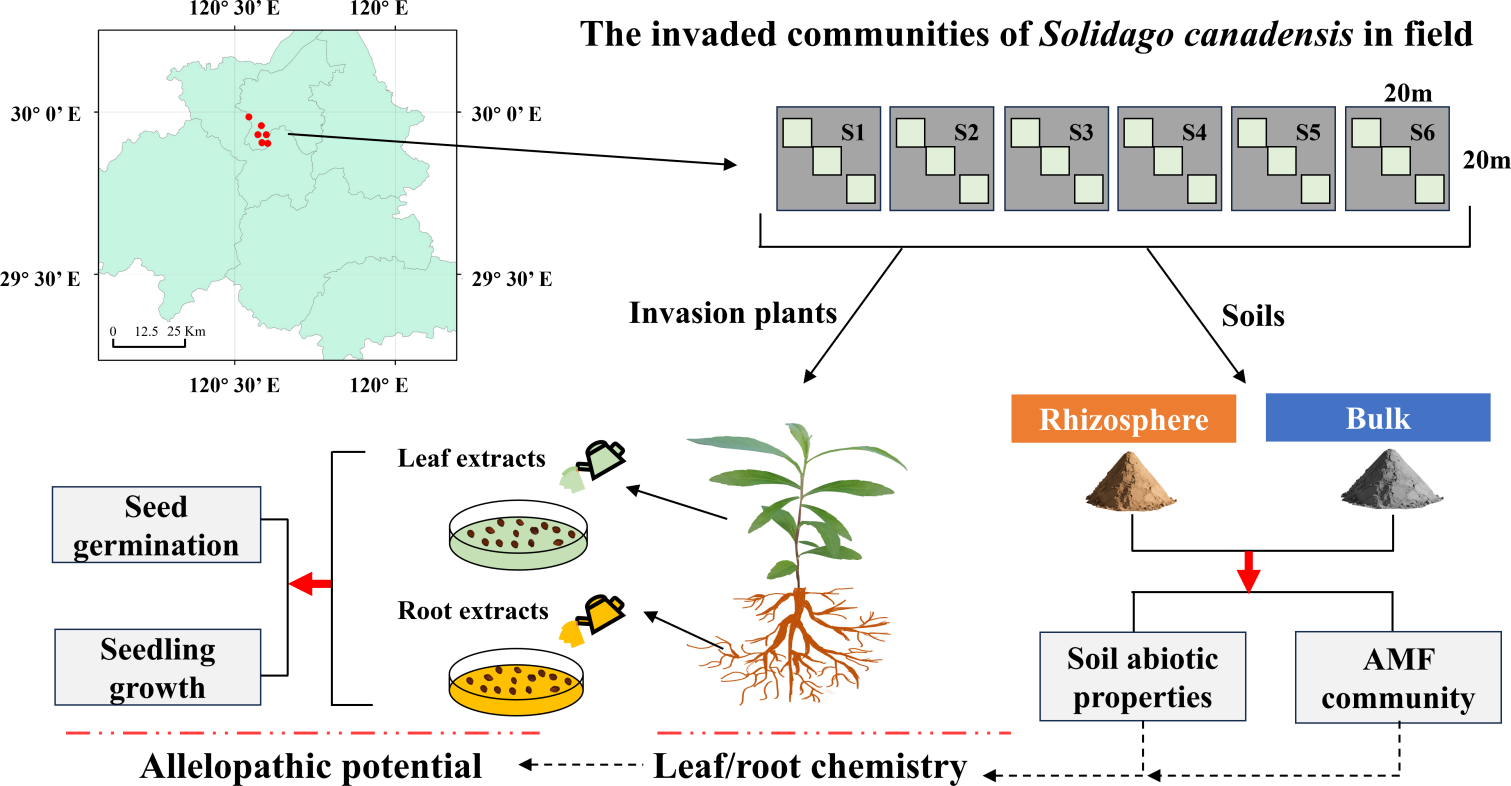
The sampling and experimental design. S1 to S6 represent six sampling sites in Shaoxing, each with three invaded quadrats by *Solidago canadensis*; in each quadrat, leaf and root tissues, and rhizosphere and bulk soils of *S. canadensis* were acquired, respectively. Then, allelochemicals of leaf and root tissues were extracted and their allelopathic potential examined. Finally, the relationships among soil factors, leaf and root chemistry, and allelopathic potential were evaluated.

Field samples were collected in Shaoxing, China, where Xu et al. (2024) compared the difference and feedback effects of AMF communities associated with *S. canadensis* and native plant species. To explore spatial variations of soil abiotic properties and AMF communities in the *S. canadensis* invasion areas, six sampling sites invaded by *S. canadensis* were first chosen. The sampling location, in the northern region of Shaoxing, Zhejiang Province, China (119°53’ ~ 121°13’ E, 29°13’ ~ 30°16’ N, **Figure 1**), has a subtropical monsoon climate. We randomly selected six sampling sites to set up *S. canadensis* plots (20 m × 20 m) and established three invaded quadrats (2 m × 2 m) per site (**Figure 1**). To minimize the likelihood of sampling the same genetic clone and to reduce spatial autocorrelation among quadrats within the same site (Eckert et al., 2021), we maintained a minimum distance of 5 m between quadrats. This spacing is expected to ensure sufficient independence of quadrat-level measurements, as it conservatively exceeds the expected root spread of *S. canadensis* and the local influence of its associated mycorrhizal hyphae. The three quadrats within each site were used as replicates for assessing site-level differences. Plant communities were characterized through systematic recording of species abundance and cover in each quadrat to evaluate invasive species competitiveness (**Supplementary Table 1**). The relative abundance of invasive *S. canadensis* was calculated as its abundance proportion to total plant abundance per quadrat, and its dominance was determined by the ratio of relative abundance to cover. Invasion intensity was used to describe the degree to which *S. canadensis* dominated each invaded quadrat, based on its relative abundance, cover, and height.

We then collected invasive plant materials and divided them into shoots and roots. Specifically, we selected the plants of *S. canadensis* with similar growth status in each invaded quadrat and collected their rhizosphere and bulk soils. In total, 18 plant samples (shoots/roots), 18 rhizosphere soil samples, and 18 bulk soil samples were acquired. Here, fresh mature leaves (fully expanded, non-senescent) and healthy roots of *S. canadensis* were exclusively sampled. We avoided senescent/abscised leaves based on their lower levels of bioactive compounds (e.g., phenols and flavonoids) compared to fresh tissues. All the leaf and root materials were washed and air-dried until processing for metabolite analyses or allelopathic bioassays, respectively. Here, although drying plant tissues may result in the breakdown or loss of some chemicals, the consistency of analysis ensures comparability across different environments (Meiners et al., 2017). All soil samples that passed through 2-mm mesh were divided into two parts: one was air-dried for physicochemical property analysis and the other was stored at -80 °C for subsequent AMF community analysis (**Supplementary Table 2**).

### Analysis of AMF community and soil abiotic properties

To determine the AMF community in soils, we extracted total soil DNA from each soil sample using an OMEGA Soil DNA Kit. DNA concentration and purity were assessed using a NanoDrop NC2000 spectrophotometer and 1.2% agarose gel electrophoresis. All DNA samples were frozen at -80 °C for further analysis. The forward primer (5’-AAGCTCGTAGTTGAATTTCG-3’) and the reverse primer (5’-CCCAACTATCCCTATTAATCAT-3’) were then used for polymerase chain reaction (PCR), and the expected amplicon length was approximately 300 bp (Sato et al., 2005). PCR amplification was performed under the following cycle parameters: 2 min at 98 °C for initial denaturation; 30 cycles of 15 s at 98 °C, 30 s at 55 °C, 30 s at 72 °C; and a final extension of 5 min at 72 °C. After electrophoresis and purification, PCR products were sequenced using an Illumina HiSeq 2500 platform at Personal Biotechnology Co., Ltd. (Shanghai, China). Further details on kits and instruments can be found in Xu et al. (2024).

The soil abiotic properties, including pH, total nitrogen (TN), total phosphorus (TP), electrical conductivity, water content, acid phosphatase (S-ACP), ammonium (NH_4_^+^-N), and nitrate (NO_3-_−N) in each quadrat were measured (see more details in Xu et al., 2024). Briefly, soil pH and electrical conductivity were determined using a pH meter and a conductivity meter, respectively. TN and the carbon–nitrogen ratio (C/N) were measured using an elemental analyzer. NH_4_^+^-N and NO_3-_−N were quantified by a Flow analyzer (Zellwegger Analytical). TP was evaluated using the molybdenum blue colorimetric method, and S-ACP was extracted using a kit (Solarbio, Beijing, China).

### HPLC-based characterization of leaf and root chemical profiles

Before the germination bioassay, root and leaf metabolites were characterized using high-performance liquid chromatography (HPLC). Briefly, in each quadrat, we pooled root or leaf tissue from five randomly selected *S. canadensis* individuals to obtain a 1 g composite sample. This sample was then ground into a fine powder and homogenized with 40 mL of distilled water. The mixture was centrifuged and filtered to obtain the supernatant, which was quantified using an Agilent 1260 Infinity Liquid Chromatography System (Agilent Technologies, CA, USA). The compounds were separated on an Ultimate LP-C18 column (250 × 4.6 mm, 5 μm, Yuexu Technology Corporation, Shanghai, China). The mobile phase consisted of 0.1% formic acid aqueous solution (A) and acetonitrile (B). The gradient elution program was set as follows: 0–10 min, 14% B; 10–11 min, 14-18% B; 11–25 min, 18% B; 25–40 min, 18-35% B; 40–45 min, 35-50% B; 45–50 min, 50-75% B; 50–55 min, 75-100% B; 55–65 min, 100% B; 65–70 min, 100-14% B. The injection volume was 20 μL, the detection wavelength was 254 nm, the column temperature was maintained at 25 °C, and the flow rate was set at 0.7 mL min^-1^. The number of chromatographic peaks was used as a descriptive indicator of chromatographic complexity under the employed HPLC conditions, allowing comparative assessment of chemical complexity across samples without inferring absolute metabolite richness (Kuhl et al., 2012).

The dried roots and leaves of *S. canadensis* from each quadrat were first mixed with deionized water (1:40, w:v) and stirred on a magnetic stirrer for 24 h at room temperature to obtain root or leaf extracts, respectively. Such water extracts generated at a biomass-to-water ratio can more accurately reflect the ecological reality of what neighboring plants and soil microbes encounter in the field and allow for differentiation among allelopathic species through altered germination of target species (Pisula and Meiners, 2010; Meiners et al., 2017). Roots were not ground, and leaves were kept whole to avoid the release of compounds that may not be produced in natural environments (Inderjit and Dakshini, 1995). The extracts were then filtered and diluted with deionized water to obtain extracts of different concentrations ranging from 0 to 100%, in 20% increments. These extracts were used to assess how plant chemistry and allelopathic potential varied with the invasive plant community and its soil environment.

To further assess the effects of allelochemicals in the extracts, total phenolic and total flavonoid contents of leaf and root extracts at 100% concentrations were also measured. Briefly, total phenolic content was determined by the Folin-Ciocalteu method, and the assay was performed according to previous literature (Singleton et al., 1999). Results were expressed as milligrams of gallic acid equivalents per gram of material (mg GAE/g). Total flavonoid content was determined by the AlCl_3_ colorimetric method (Jia et al., 1999), and the results were expressed as milligrams of rutin equivalents per gram of dry weight (mg RE/g).

### Bioassay for allelochemicals: seed germination and seedling growth

*R. sativus* seeds were surface-sterilized with 2% sodium hypochlorite (NaClO) for 10 min and washed three times with distilled water before being placed in Petri dishes (90 mm in diameter). In total, 20 seeds were placed on sterilized filter paper per dish to evaluate germination. Each dish received 4 mL of filtered extract at five concentrations (20, 40, 60, 80, and 100%) or distilled water as a control. In total, there were 186 dishes: 18 invaded communities × 2 extracts (leaf and root) × 5 concentration treatments + 6 controls. All Petri dishes were wrapped in zip-lock bags to maintain moisture and placed in an incubator at 25 °C with a 12 h light/12 h dark photoperiod. Germination was recorded daily until germination count stabilized. To further evaluate the effect of extracts on seedlings, all germinated seedlings in each dish were cultured for another two weeks and finally the mean fresh weight, hypocotyl length, and root length were measured.

### Data analysis

To compare the effects of leaf and root extracts on seed germination and seedling growth (including germination rate, seedling fresh weight, hypocotyl length, and root length) relative to the control, a one-way analysis of variance (ANOVA) with Dunnett’s *post hoc* test was performed, with treatment (control, leaf extract, and root extract) as the fixed factor. Leaf and root extract data were averaged across the three quadrats within each site to obtain site-level means, while the control consisted of six independent replicates and served only as a common baseline for bioassay comparison.

Two-way analysis of variance with Tukey’s *post hoc* multiple comparisons was used to analyze differences in seedling hypocotyl length, total phenolics, total flavonoids, and chromatographic peak number, with site and organ (leaf and root) as fixed factors. To compare the overall allelopathic strength between leaf and root extracts across all sites, one-way analysis of variance (ANOVA) was performed on germination rate and seedling growth parameters, with extract organ (leaf vs. root) as fixed factors. The relationships between total flavonoids or total phenolics and seed germination or seedling growth parameters were evaluated using Pearson correlation analysis.

To characterize AMF community composition, cluster analysis and taxonomic analysis were performed based on operational taxonomic unit (OTU) tables. Nonmetric multidimensional scaling (NMDS) ordination and permutational multivariate analysis of variance (PERMANOVA) based on Bray-Curtis distance matrices were conducted to compare AMF community composition between bulk and rhizosphere soils. Differences in AMF community richness and alpha-diversity indices (Shannon-Wiener, Pielou evenness, Chao1, and ACE indices) among field soils were assessed using the *vegan* package. To compare major allelochemicals between roots and leaves, principal component analysis (PCA) was performed on feature peaks (chromatographic peaks exceeding 10 mAU·s) using the *FactoMineR* package, and results were visualized using the *factoextra* package. The *psych* and *dplyr* packages were employed to analyze correlations among flavonoids, phenolic compounds, chromatographic peak number, PC1/PC2 of leaf/root tissues, and 10 dominant AMF genera. Associations among leaf/root extracts’ PC axes, rhizosphere and bulk soil abiotic properties, and AMF properties were analyzed using Mantel tests implemented in the *vegan* package with 9,999 permutations. Finally, a hypothesized pathway linking plant, soil, and allelopathic potential was constructed using partial least squares path modeling (PLS-PM) with the *plspm* and *devtools* packages. All data processing was performed in R 4.3.3.

## Results

### Inhibitory effects of allelochemicals and their spatial variation

**Figure 2:**
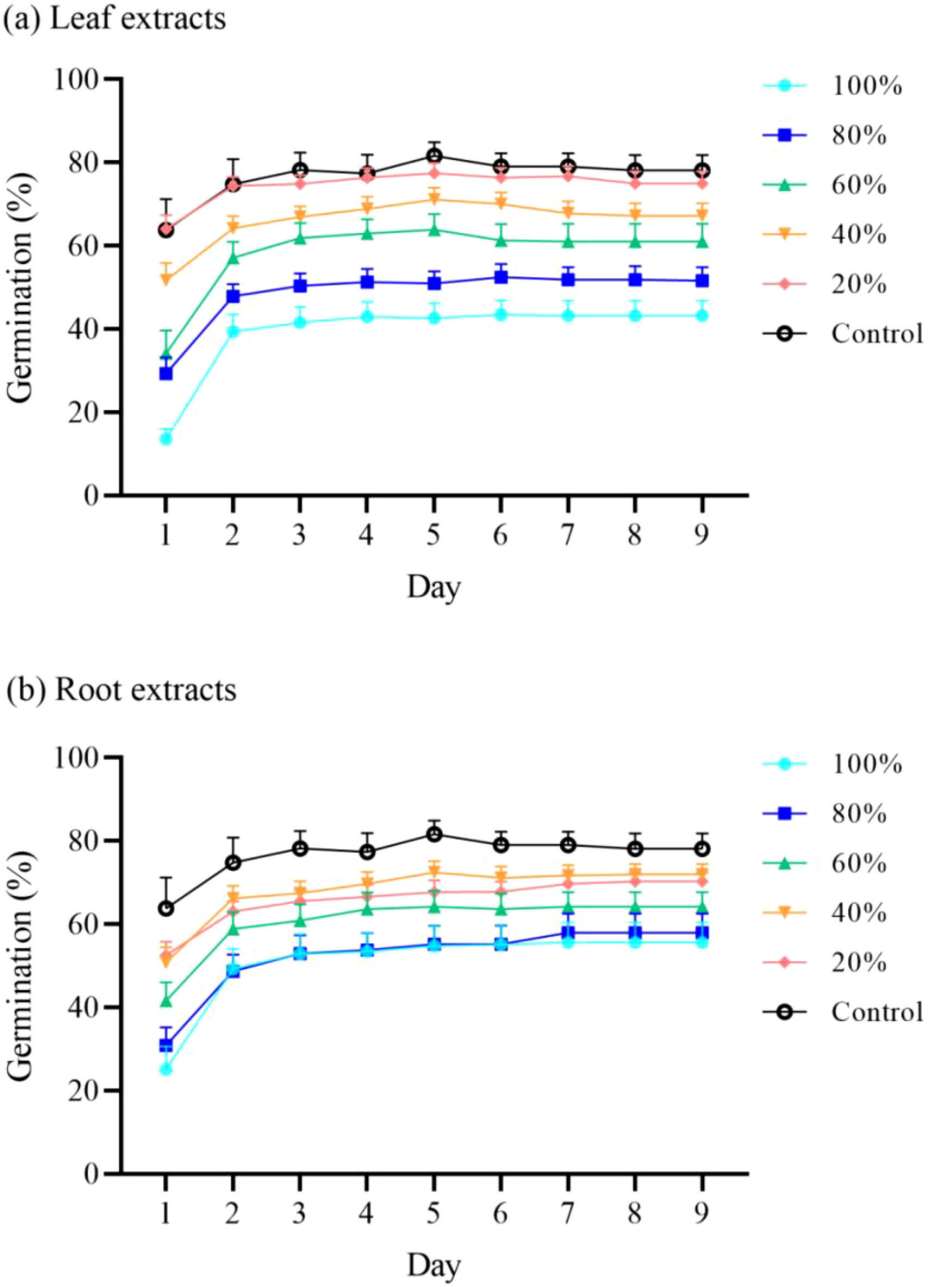
Effects of *S. canadensis* leaf **(a)** and root **(b)** extracts at six concentrations on *R. sativus* seed germination. Data are means ± SE (n = 18, six sites × three quadrats).

**Figure 3:**
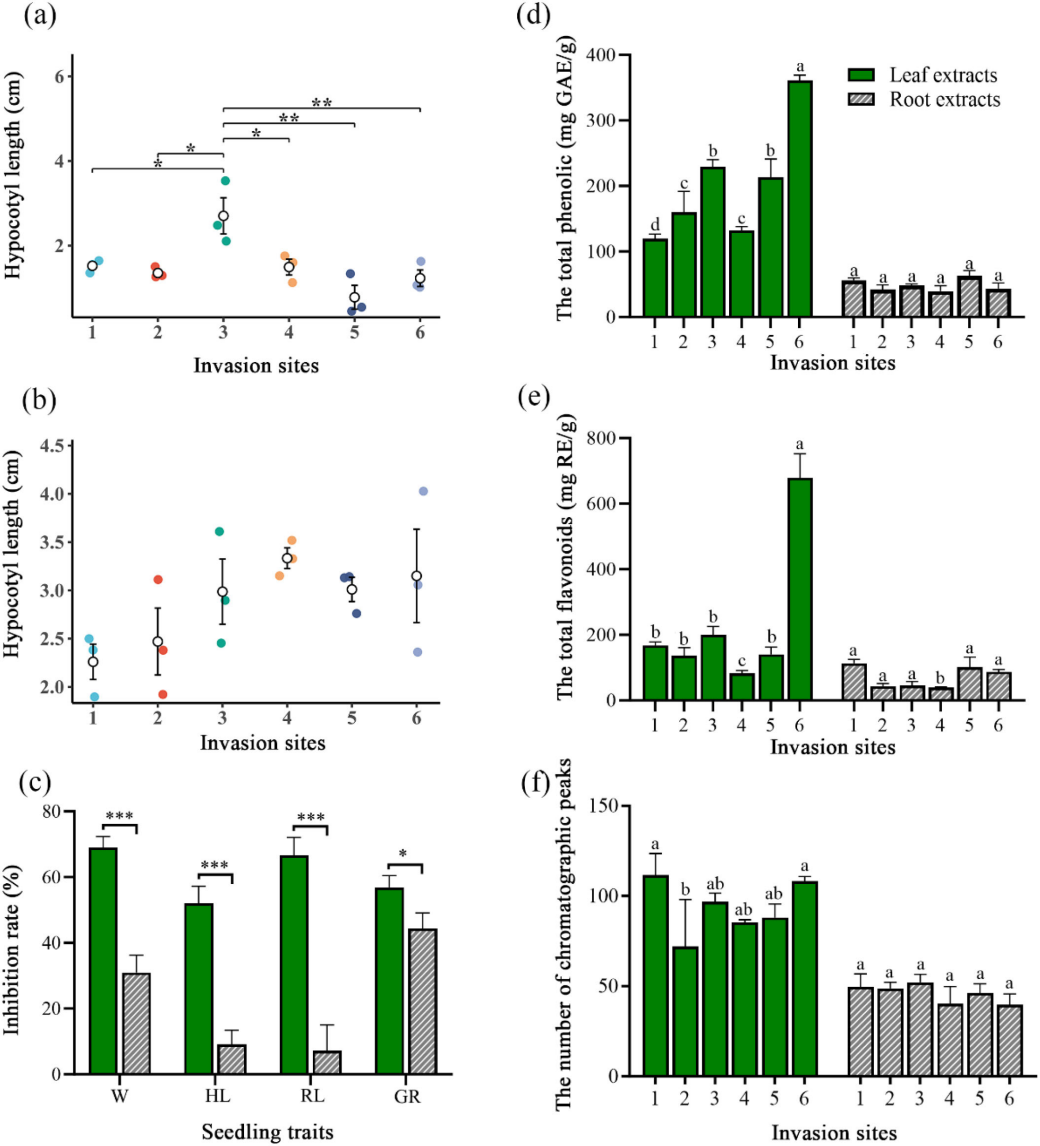
Allelopathic effects of **(a)** leaf and **(b)** root extracts of *S. canadensis* collected from six sites on the hypocotyl length of *R. sativus;* **(c)** inhibitory effects of leaf and root extracts on seed germination and seedling growth of *R. sativus.* Differences in **(d)** total phenols, **(e)** total flavonoids, and **(f)** the number of chromatographic peaks of leaf and root extracts at six sites. W: seedling fresh weight; HL: seedling hypocotyl length; RL: seedling root length; GR: seed germination rate. For panels **(a, b, d–f)**, values are means ± SE for three quadrats within each site (n = 3). For panel **c**, values are means ± SE based on all sampled extracts from the six sites (n = 18). Different letters indicate significant differences. ***, **, and * indicate the significance at levels of 0.001, 0.01, and 0.05, respectively.

The germination and seedling growth of *R. sativus* decreased as leaf and root extract concentrations increased (**Figure 2**; **Supplementary Figure 1**). At the highest concentration tested (100%), both leaf and root extracts of *S. canadensis* significantly affected germination and seedling growth of *R. sativus* (**Table 1**). Compared with the control, the leaf extract significantly inhibited seed germination, seedling weight, hypocotyl length, and root length (*P* < 0.05), while the root extract significantly inhibited seed germination and seedling weight (*P* < 0.05, **Table 1**). Simultaneously, the negative allelopathic strength of *S. canadensis* showed notable spatial heterogeneity across various invasion sites (**Figures 3a, b**; **Supplementary Table 3**). Hypocotyl length showed significant effects of site (*F_(5, 24)_*= 4.01, *P* = 0.0087), organ (*F_(1, 24)_*= 75.29, *P* < 0.001), and their interaction (*F_(5, 24)_*= 3.98, *P* = 0.0091, **Table 2**). Under leaf extracts, hypocotyl length at site 3 was significantly higher than at sites 1, 2, 4, 5, and 6, whereas root extracts showed weaker variation among sites (**Figures 3a, b**).

**Table 1:** Effects of 100% leaf and root extracts of *S. canadensis* on seed germination and seedling growth of *R. sativus*.

Treatment	Seed germination (%)	Seedling weight (g)	Hypocotyl length (cm)	Root length (cm)
Leaf extracts	43.20 ± 5.40***	0.57 ± 0.09***	1.51 ± 0.26***	1.21 ± 0.25***
Root extracts	55.66 ± 4.16**	1.27 ± 0.08***	2.87 ± 0.17	3.38 ± 0.22
Control	78.15 ± 3.64	1.84 ± 0.92	3.16 ± 0.17	3.63 ± 0.21

Data are presented as mean ± SE (n = 6). Significance levels compared to the control: *P < 0.05, **P < 0.01, ***P < 0.001.

**Table 2:** Two-way ANOVA results for the effects of site, organ (leaf and root), and their interaction on hypocotyl length, total phenolic content, total flavonoid content, and the number of chromatographic peaks.

Fixed effects	Total phenolic content			Total flavonoid content			Number of chromatographic peaks			Hypocotyl length		
	*df*	*F*	*P*	*df*	*F*	*P*	*df*	*F*	*P*	*df*	*F*	*P*
Site	5	40.19	**< 0.001**	5	114.06	**< 0.001**	5	3.80	**0.011**	5	4.01	**0.008**
Organ	1	717.76	**< 0.001**	1	329.44	**< 0.001**	1	213.69	**< 0.001**	1	75.29	**< 0.001**
Sites × organ	5	49.03	**< 0.001**	5	97.54	**< 0.001**	5	4.07	**0.008**	5	3.98	**0.009**

Significant effects (*P* < 0.05) are bold (n = 3).

ANOVA, analysis of variance.

The inhibitory effect of leaf extracts was higher than that of root extracts on germination of *R. sativus* (*F_(1, 34)_* = 4.345, *P* = 0.045), and on its seedling growth parameters, including seedling weight, hypocotyl length, and root length (*F_(1, 34)_* = 37.195, *P* < 0.001; *F_(1, 34)_* = 40.043, *P* < 0.001; *F_(1, 34)_* = 38.367, *P* < 0.001, **Figure 3c**). Furthermore, total phenolics (*F_(5, 24)_*= 40.19, *P* < 0.001, **Figure 3d**), total flavonoids (*F_(5, 24)_* = 114.06, *P* < 0.001, **Figure 3e**), and the number of chromatographic peaks (*F_(5, 24)_* = 3.80, *P* = 0.011, **Figure 3f**) all varied significantly among sites (**Table 2**). Notably, correlation analyses further revealed that phenols showed significantly greater inhibitory effects on both seed germination and seedling growth of *R. sativus* compared to flavonoids (**Table 3**). In total, all allelochemicals, including phenols, flavonoids, and chromatographic peak numbers, were also more abundant in leaf extracts than in root extracts (*F_(1, 24)_*= 717.76, *P* < 0.001; *F_(1, 24)_* = 329.44, *P* < 0.001; *F_(1, 24)_* = 213.69, *P* < 0.001, **Figures 3d–f**). These differences further depended on both site and organ, as indicated by the significant interactive effects of the two factors on total phenolics (*F_(5, 24)_* = 49.03, *P* < 0.001, **Table 2**), total flavonoids (*F_(5, 24)_* = 97.54, *P* < 0.001, **Table 2**), and the number of chromatographic peaks (*F_(5, 24)_* = 4.07, *P* = 0.008, **Table 2**).

**Table 3:** Correlations between total phenols/flavonoids of *S. canadensis* and seed germination and seedling growth of *R. sativus*.

Seedling traits	Total phenol content (mg GAE/g)	Total flavonoid content (mg RE/g)
Germination	-0.332 (0.048*)	-0.179 (0.296)
Weight	-0.609 (< 0.001***)	-0.393 (0.018*)
Hypocotyl length	-0.590 (< 0.001***)	-0.390 (0.019*)
Root length	-0.540 (< 0.001***)	-0.397 (0.017*)

Correlations were calculated using pooled leaf and root observations across all sampled quadrats. Significance levels: *P < 0.05, **P < 0.01, ***P < 0.001.

### Associations among AMF, soil abiotic factors, and allelochemicals

The PCA results showed that the first two principal components explained 49.7% of the total variance in leaf extracts and 42.2% in root extracts (**Supplementary Figure 2**). Several chromatographic features contributed strongly to PC1 and PC2 (**Supplementary Figure 3**). Based on their UV absorption at 254 nm, retention behavior, comparison with previously reported phytochemical profiles of other plants, and concordance with total phenolic and flavonoid contents, the representative compounds were mainly flavonoids and phenols (**Supplementary Table 4**).

**Figure 4:**
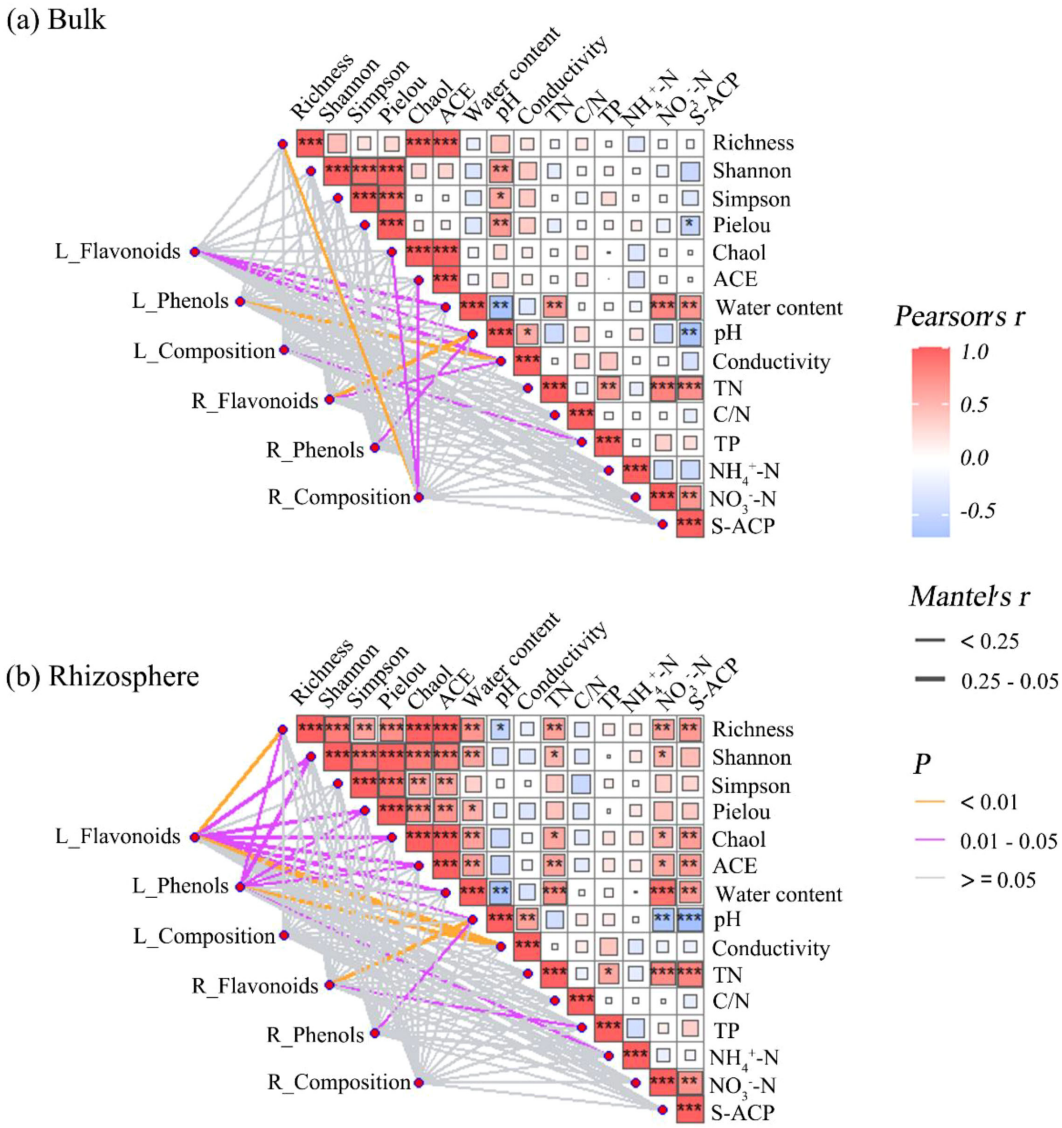
Mantel test analysis of soil abiotic factors and arbuscular mycorrhizal fungal (AMF) communities in relation to root/leaf allelochemicals. Mantel test chart for **(a)** bulk and **(b)** rhizosphere soil. L and R represent leaves and roots, respectively. Pielou, Pielou’s evenness index; ACE, Abundance-based Coverage Estimator; TN, total nitrogen; C/N, C:N ratio; TP, total phosphorus content; S-ACP, soil acid phosphatase. *P < 0.05, **P < 0.01, ***P < 0.001.

Mantel tests revealed associations of leaf/root chemistry with soil abiotic properties and AMF communities. As shown in **Figure 4**, in bulk soils, flavonoids and phenols in leaves and roots showed significant correlations with soil water content, pH, electrical conductivity, and total P (all *P* < 0.05; **Figure 4a**). Root allelochemical composition showed marked associations with AMF richness, Chao1, and ACE (Abundance-based Coverage Estimator) indices (all *P* < 0.05; **Figure 4a**). In rhizosphere soils, flavonoids and phenols in leaf extracts exhibited stronger correlations with AMF richness, Shannon, Pielou, Chao1, and ACE indices, but weaker correlations with water content, pH, and electrical conductivity. In contrast, root flavonoids only correlated with soil pH and TP, and root phenols showed pH-specific associations (all *P* < 0.05; **Figure 4b**). However, the overall allelochemical composition (represented by PCA axes) of both leaves and roots showed no significant correlation with any environmental factors in the rhizosphere (**Figure 4b**).

**Figure 5:**
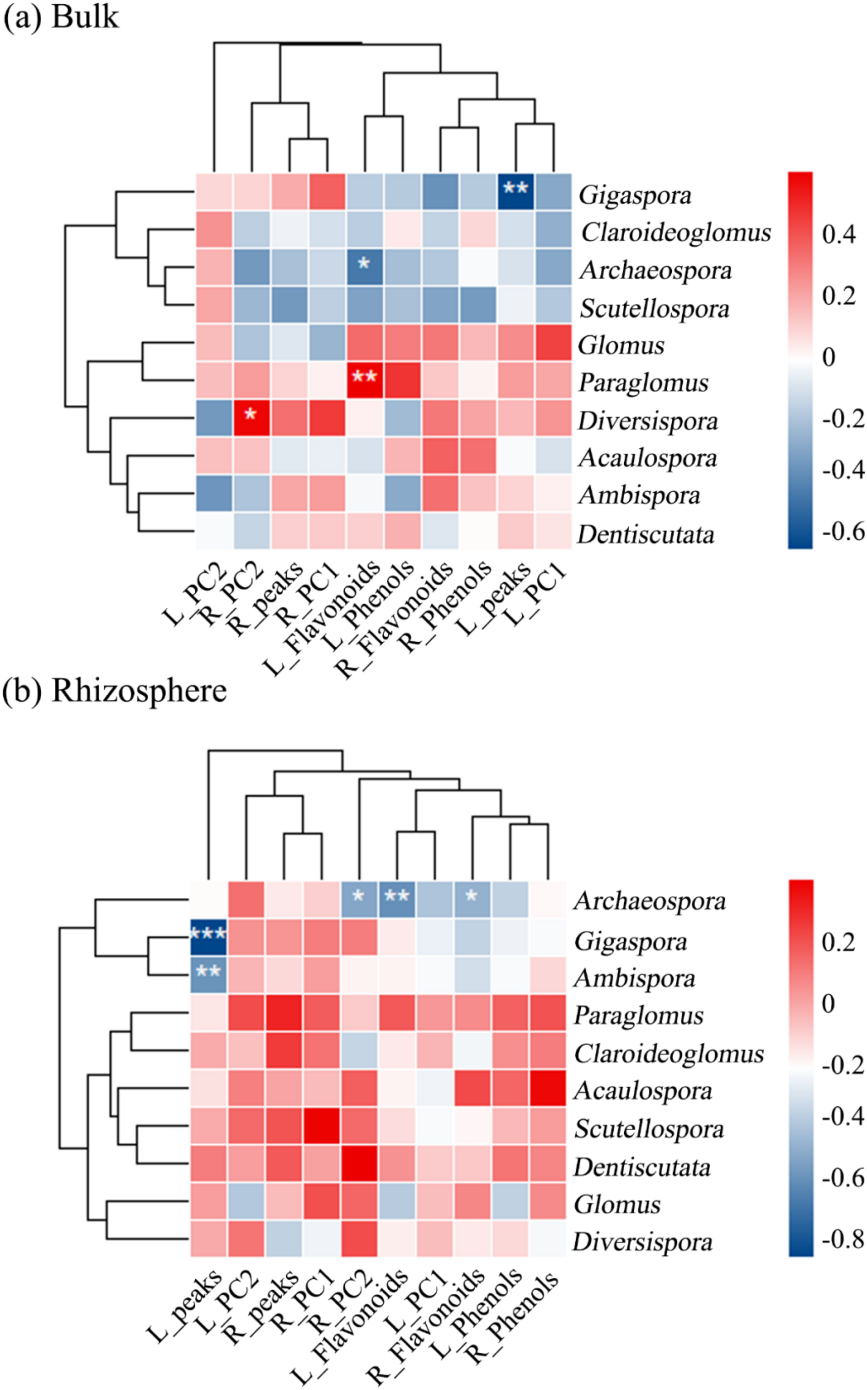
Correlations between allelochemical traits of *S. canadensis* and the dominant genera in arbuscular mycorrhizal fungal (AMF) communities in bulk **(a)** and rhizosphere **(b)** soils. L and R represent leaves and roots, respectively. Peaks indicate the number of chromatographic peaks, a measure of chromatographic complexity. PC1 and PC2 indicate the first two principal components derived from PCA of feature peaks. *P < 0.05, **P < 0.01, ***P < 0.001.

Here, AMF Shannon diversity and Pielou evenness in rhizosphere soils differed from those in bulk soils (**Supplementary Figure 4a**; *P* < 0.05). The community composition in the two soils was clearly different (**Supplementary Figures 4b, c**). Bulk soil *Paraglomus* abundance was positively associated with leaf flavonoid contents (L_flavonoids), while *Gigaspora* and *Archaeospora* were negatively correlated with leaf chromatographic complexity (L_peaks) and flavonoid contents (**Figure 5a**). In addition, the root PC2 (R_PC2) axis was positively associated with *Diversispora* (**Figure 5a**). In the rhizosphere, flavonoids in both leaf and root extracts (L_flavonoids and R_flavonoids) were negatively correlated with *Archaeospora* (**Figure 5b**). Both *Ambispora* and *Gigaspora* were negatively associated with leaf chromatographic complexity (L_peaks) (**Figure 5b**, all *P* < 0.05).

### Integrated associations among AMF, soil factors, allelochemicals, and allelopathic potential

**Figure 6:**
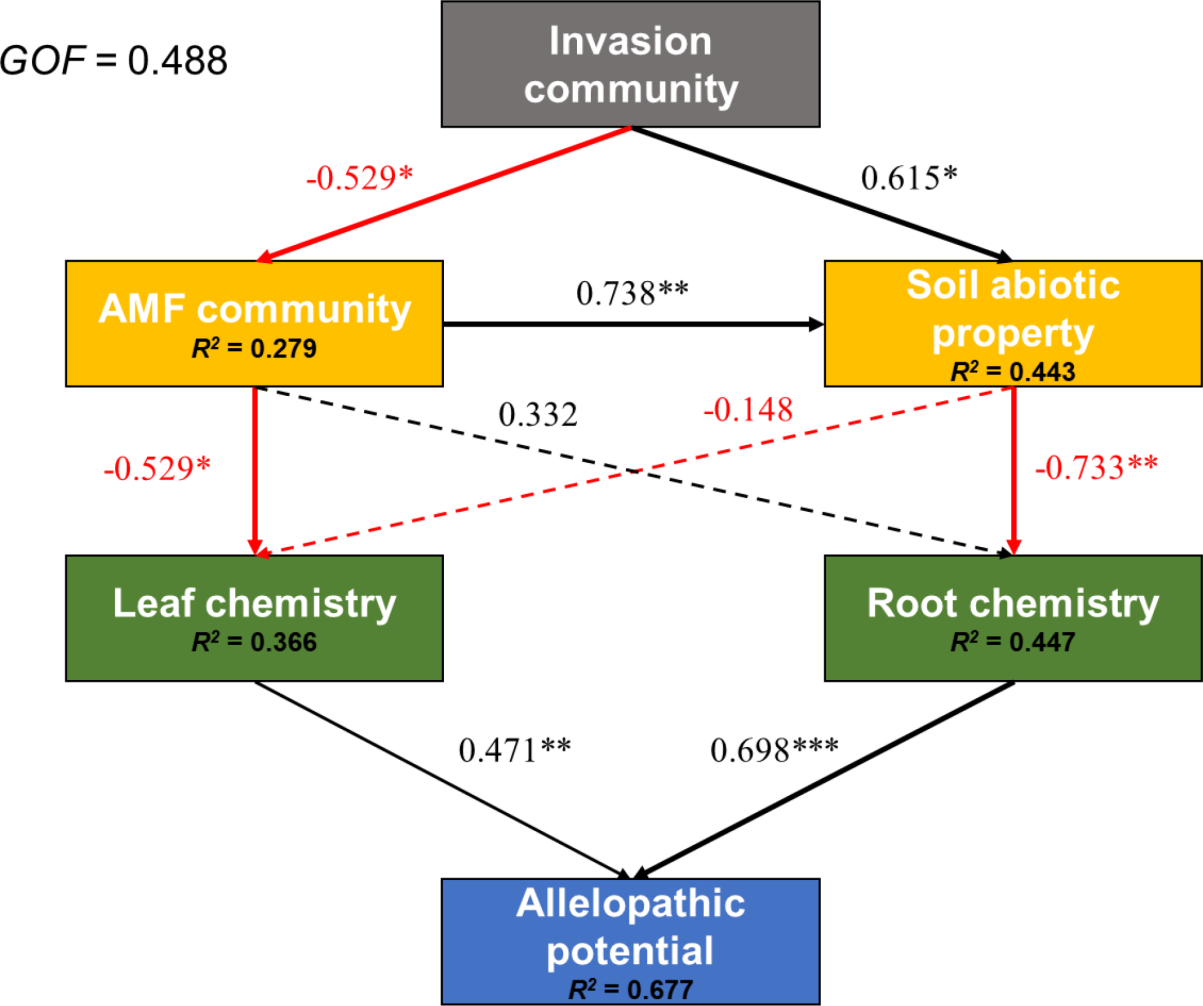
Cascading associations among invasion community (relative abundance, cover), soil abiotic property (pH, NO3--N, and S-ACP), arbuscular mycorrhizal fungal (AMF) community (Shannon, Pielou, and Chao1 indices), leaf chemistry (total flavonoid content and total phenol content), root chemistry (total flavonoid content, total phenol content and chromatographic peak numbers) and allelopathic potential (inhibitory rates of root and leaf extract treatments on seedling fresh weight, hypocotyl length, and root length). Red and black lines denote positive and negative path coefficients, respectively. Dashed lines represent the significance at *P* > 0.05. Numbers on arrow lines represent significant standardized path coefficients. *P < 0.05, **P < 0.01, ***P < 0.001. *R2* values represent the variance of dependent variables explained by the inner model.

The PLS-PM model showed an overall acceptable fit (GOF = 0.488; **Figure 6**). The measurement model showed satisfactory quality for the reflective latent variables, with acceptable composite reliability and average variance extracted for the Invasion community, AMF community, leaf chemistry, and root chemistry, whereas soil abiotic property and allelopathic potential were specified as formative constructs. The structural model explained a moderate proportion of variance in the AMF community (*R^2^* = 0.279), soil abiotic property (*R^2^* = 0.443), leaf chemistry (*R^2^* = 0.366), and root chemistry (*R^2^* = 0.447), and a relatively high proportion of variance in allelopathic potential (*R^2^* = 0.677). Complete model diagnostics, including cross-loadings and bootstrap validation, are provided in **Supplementary Table 5**.

All path coefficients reported below are standardized coefficients (β) from the PLS-PM model. In the field, the invasion community tended to be negatively associated with the AMF community (β = -0.529, *P* = 0.024) but positively associated with soil abiotic property (β = 0.615, *P* = 0.016). The AMF community showed a positive association with soil abiotic property (β = 0.738, *P* = 0.005) and a negative association with leaf chemistry (β = -0.529, *P* = 0.033). By contrast, root chemistry was not significantly related to the AMF community (β = 0.332, *P* = 0.136) but showed a negative association with soil abiotic property (β = -0.733, *P* = 0.003). At the downstream end of the model, allelopathic potential was positively associated with both leaf chemistry (β = 0.471, *P* = 0.006) and root chemistry (β = 0.698, *P* < 0.001). Overall, the PLS-PM analysis suggested a correlative framework in which the Invasion community, AMF community, soil abiotic property, and plant chemical traits were interconnected and jointly associated with spatial variation in allelopathic potential.

## Discussion

Our study provides direct field evidence that the allelopathic potential of invasive *S. canadensis* is a spatially structured trait intimately linked to the belowground environment. The findings strongly support our three predictions: significant spatial variation in leaf and root chemistry (H1), systematic correlations of this variation with both AMF community composition and key soil abiotic properties (H2), and functional divergence in allelopathic strength between plant organs (H3). Crucially, by establishing these correlations in natural invasion contexts, our work addresses a key knowledge gap: how PSI-related factors covary with allelopathic expression in the field. These results shift the perspective on the AMF’s role from that of a passive target of allelochemicals to an integral component of the soil environment whose variation is associated with the plant’s chemical interference strategy. This field-observed pattern provides an empirical basis for future mechanistic studies.

In general, allelopathic effects in the field can vary widely with plant traits, experimental approaches, chemical sources, exposure duration, chemical identity and concentration, phylogenetic distance, and soil context (e.g., microbes and nutrients) (Meiners et al., 2017; Zhang et al., 2021; Cai et al., 2024). In our study, the contrasting community composition across sites (**Supplementary Table 1**) provides a compelling, site-specific context for this variation. For instance, sites 4 and 6, characterized by very high relative abundance of *S. canadensis* (74.21% and 72.97%, respectively), may represent advanced invasion stages where strong, invader-dominated plant-soil feedbacks have been established (Pendergast et al., 2013; Meiners et al., 2017; Judzentiene et al., 2023). Conversely, a site such as Site 3, with much lower invader abundance (22.52%), might reflect an earlier invasion phase or a more resistant community with different soil legacies. Consistently, the inhibition of hypocotyl length appeared to be consistent with invasion intensity. Thus, our data outline a correlative framework in which the gradient in invasion intensity is closely linked to distinct local soil environments, which in turn are associated with variation in allelochemical production and potency.

Interestingly, distinct associations emerged between AMF community attributes and the allelochemical profiles of *S. canadensis* leaves and roots. Root allelochemicals (particularly flavonoids and phenols) were more strongly linked to AMF richness in bulk soils, whereas leaf allelochemicals showed tighter correlations with multiple AMF diversity indices in the rhizosphere. This organ-specific pattern suggests that leaves and roots may engage with different aspects of the AMF community. Furthermore, the spatial order of AMF diversity across invasion sites paralleled the gradient in leaf extract inhibition strength, consistent with a positive association between rhizosphere AMF diversity and aboveground allelopathic expression. These field-observed associations align with mechanistic insights from controlled studies. Research suggests that AMF can influence host plant secondary metabolism through various pathways, such as releasing signaling molecules (e.g., strigolactones) or altering carbon and nutrient allocation (Smith and Smith, 2011; Lanfranco et al., 2018). For example, specific AMF taxa such as *Rhizophagus irregularis* have been shown to increase flavonoid and phenolic production in some plants (Xie et al., 2018; Lv et al., 2024), while *Glomus mosseae* and *G. intraradices* enhanced phenolic compound accumulation in *Trifolium repens* and *Echinacea purpurea*, respectively (Araim et al., 2009; Zhang et al., 2013). Our findings extend this knowledge by demonstrating taxon-specific association patterns in a field setting. We found that *Paraglomus* in bulk soil was positively correlated with leaf flavonoids, whereas *Archaeospora* and *Gigaspora* (in both soil compartments) were negatively correlated with allelochemical measures. Collectively, these results imply that variation in AMF community composition (both in overall diversity and in the abundance of specific taxa) is associated with distinct expression of plant allelochemicals (Lv et al., 2024). This supports the prediction that AMF communities are not passive bystanders but may be linked to variation in biochemical pathways underlying the invasive plant’s chemical defense and its spatial variation.

Apart from AMF, several soil abiotic properties, including water content, pH, electrical conductivity, TP, and NH_4_^+^-N, showed significant associations with the chromatographic complexity and concentration of allelochemicals in *S. canadensis* leaves and roots. This aligns with the understanding that abiotic factors can trigger plants to produce specific allelochemicals, thereby influencing neighboring organisms (Shan et al., 2023). First, soil pH and ionic strength not only mediate AMF communities (Xu et al., 2024), but they also regulate secondary metabolites, including leaf/root phenols and flavonoids (Li et al., 2010; Radic et al., 2016), both of which may directly and indirectly affect the allelopathic potential of *S. canadensis*. Furthermore, nutrient availability is another key abiotic correlate. For example, nitrogen can modulate the accumulation of carbon-based secondary metabolites such as glycyrrhizin (Heimler et al., 2017; Xie et al., 2018; Yu et al., 2020). Our data specifically linked higher rhizosphere NH_4_^+^-N to increased leaf phenol content, a pattern that is consistent with amplified carbon flux into phenylpropanoid pathways following enhanced NH_4_^+^-N uptake via AMF hyphae (Lv et al., 2024). Interestingly, leaf chromatographic complexity was negatively correlated with total phosphorus, implying that phosphorus availability may constrain the allelopathic potential of invasive plants, possibly due to a trade-off between allelochemical production and phosphorus-scavenging investments (e.g., phosphatase secretion) (Chen et al., 2023). Collectively, these abiotic associations underscore that the soil’s physicochemical environment is integral to the context-dependent expression of allelopathy of *S. canadensis*.

The PLS path model summarized a network of statistical associations in which greater invasion intensity was linked to distinct AMF communities and soil properties, which were in turn correlated with changes in leaf and root chemistry related to allelopathic potential. This correlative framework aligns with the plant-soil feedback hypothesis, where invasive plants are associated with modified soil biota that may favor their own growth (Callaway et al., 2004; Xu et al., 2024). The divergent associations for leaf versus root chemistry suggest a possible organ-specific resource allocation pattern. In this pattern, invasion-linked AMF communities might be associated with enhanced production of leaf defense compounds (e.g., phenolics; Koricheva et al., 2009) while showing a different relationship with root carbon allocation-a configuration that could, in theory, maximize allelopathic impact while minimizing costs (Pendergast et al., 2013; Wang et al., 2022; Fadiji et al., 2025). Consistent with this, and with prior reports (Kato-Noguchi and Kato, 2022; Perera et al., 2023), we found that leaf extracts of *S. canadensis* exerted stronger allelopathic inhibition than root extracts. Such above-belowground divergence in metabolite allocation is a known plant strategy for adapting to heterogeneous environments (Chomel et al., 2016; Hagedorn et al., 2019; Qi et al., 2020; Bi et al., 2024). Importantly, the stronger allelopathic effect of leaves in our bioassay does not imply that roots had a weaker communication function than leaves to alter plant-plant interactions. Roots undoubtedly engage in complex belowground communication via exudates that shape the rhizosphere microbiome and influence plant-plant interactions through mechanisms such as chemical defense and nutrient transfer (Martin and Mallik, 2021).

In this study, we employed a standardized germination bioassay using a sensitive target species (*R. sativus*) to assess the allelopathic potential of *S. canadensis* across multiple field sites. This controlled approach was essential for quantifying and comparing spatial variation in allelopathic inhibition across complex natural invasion gradients. Given the observational and cross-sectional nature of our design, the associations we document between soil factors (AMF communities and abiotic properties) and allelopathic outcomes are correlative. They highlight AMF and soil properties as key, covarying components of the plant-soil environment linked to allelopathic expression, rather than as isolated causal drivers. Although our study included six invaded sites, this relatively limited number of sites restricts the broader generalizability of our findings. Furthermore, with only three quadrats sampled per site, our ability to capture fine-scale spatial variation within individual sites may be constrained. Therefore, the patterns observed here should be considered as foundational field evidence, which requires further validation through expanded sampling across a larger number of sites. Our findings underscore the pronounced context-dependency of allelopathy in the field, consistent with the conceptual framework of Meiners et al. (2017). To move from documenting patterns to unraveling mechanisms, future research should focus on the following: (1) isolating and verifying the specific allelochemicals involved in these interactions and (2) experimentally testing the functional roles of identified AMF taxa and abiotic factors. The integration of plant metabolic genomics with *in situ* chemical detection techniques will be particularly powerful for elucidating allelopathic processes within the dynamic PSI framework. Ultimately, this study provides the essential field-observed correlative landscape that defines the targets and context for such future mechanistic investigations.

## Data Availability

The raw data supporting the conclusions of this article will be made available by the authors, without undue reservation.
