# Supplementary material for: Spatial variation in AMF communities and soil properties correlates with leaf and root allelopathic potential in Solidago canadensis in six different locations
Source: Front Plant Sci. 2026 May 7;17:1755243. doi: 10.3389/fpls.2026.1755243 (PMC13190382; doi:10.3389/fpls.2026.1755243)
Supplement: Supplementary file 1 [file Supplementaryfile1.doc]

**Spatial variation in AMF communities and soil properties correlates with leaf and root allelopathic potential in *Solidago canadensis* in six different locations**

*Huan Mei^1^, Xueyi Huang^1^, Zuofu Wei^2^, Lifu Sun^1^, Huanyi Yang^1^, Xiaodong Zhang^1*^, Lijia Dong^1^**

1. *School of Life and Environmental Sciences, Shaoxing University, Huancheng West Road 508, Shaoxing, 312000, P.R. China.*
2. *School of Life Science, Shanxi Normal University, Linfen, China*

^*^Corresponding authors. Address: School of Life and Environmental Sciences, Shaoxing University, Huancheng West Road 508, Shaoxing, 312000, P.R. China

*E-mail address*: [13676885816@163.com](mailto:13676885816@163.com,); [Donglijia@126.com](mailto:Donglijia@126.com)

**Table S1 The community characteristics of invasive *S. canadensis* populations at six study sites.**

| Sites | Relative abundance (%) | Cover (%) | Height (cm) |
| --- | --- | --- | --- |
| 1 | 12.18 ± 0.03 | 71.67 ± 0.11 | 126.67 ± 19.52 |
| 2 | 20.93 ± 0.07 | 83.33 ± 0.05 | 182.33 ± 6.87 |
| 3 | 22.52 ± 0.01 | 83.33 ± 0.06 | 170.00 ± 21.60 |
| 4 | 74.21 ± 0.03 | 75.00 ± 0.05 | 190.00 ± 0.00 |
| 5 | 30.86 ± 0.03 | 80.00 ± 0.05 | 210.00 ± 4.71 |
| 6 | 72.97 ± 0.02 | 96.00 ± 0.05 | 165.33 ± 6.13 |
| Average | 38.95 | 81.56 | 174.06 |

**Table S2** Taxonomic composition and variation of AMF OTUs detected at six invasion sites

| Site | Phylum | Class | Order | Family | Genus | Species |
| --- | --- | --- | --- | --- | --- | --- |
| 1 | 120 | 73 | 84 | 2 | 301 | 142 |
| 2 | 130 | 76 | 92 | 2 | 346 | 125 |
| 3 | 112 | 50 | 69 | 1 | 232 | 91 |
| 4 | 176 | 132 | 171 | 2 | 427 | 130 |
| 5 | 151 | 99 | 132 | 2 | 401 | 152 |
| 6 | 64 | 35 | 32 | 0 | 163 | 120 |
| *P* | 0.016* | 0.007** | 0.000*** | 0.093 | 0.019* | 0.538 |

Note: ***、**、*represent the significant difference at the level of 0.001, 0.01 and 0.05.

Table S3 Chemistry variation in leaves and roots of *Solidago canadensis* across invasion sites. CV: coefficient of variance (%). Max/Min: ratio of maximum to minimum values for each chemical parameter.

| Chemicals | Mean ± se | Maximum | Minimum | Max/Min | CV% | F | *P* |
| --- | --- | --- | --- | --- | --- | --- | --- |
| leaf peak number (HPLC) | 93.667 | 119.000 | 42.000 | 2.833 | 18.629 | 4.430 | **0.016** |
| leaf flavonoids | 234.377 | 740.000 | 75.830 | 9.759 | 89.585 | 120.439 | **< 0.001** |
| leaf phenols | 202.466 | 367.730 | 352.270 | 1.044 | 42.023 | 68.347 | **< 0.001** |
| root peak number (HPLC) | 46.111 | 57.000 | 33.000 | 1.727 | 15.369 | 1.985 | 0.153 |
| root flavonoids | 71.403 | 135.420 | 30.210 | 4.483 | 46.798 | 13.861 | **< 0.001** |
| root phenols | 48.697 | 68.330 | 31.670 | 2.158 | 21.260 | 5.451 | **0.008** |

Table S4 The potential compounds identified at distinct retention time in leaf and root extracts via HPLC methods.

| Retention time | Potential compounds | Class | Reference |
| --- | --- | --- | --- |
| 3.2 | Chlorogenic acid | Phenolic acid | Apati et al. 2002  Zekic et al. 2021 |
| 5.8 | Caffeic acid | Phenolic acid | Apati et al. 2002 |
| 8.0  8.3  8.8  8.9 | Kaempferol-3-O-α-L- rutinoside (nicotiflorin)*  Chlorogenic acid, 4-O-Caffeoylquinic acid, Caffeoylshikimic acid  Cinnamic acid  Quercetin-(pentosyl)-hexoside | Flavonoids, Phenolic acids  Phenolic acids  Flavonoids | Apati et al. 2002  Judžentienė et al 2023  Zekic et al. 2021 |
| 9.2,  9.5 | Quercetin-(rhamnosyl)-hexoside,  Rutin | Flavonoids | Zekic et al. 2021 |
| 10.3,  10.8，  10.0，  10.1  10.7 | Hyperoside, Isoquercitrin,  Quercetin-3-O-β-D-rutinoside (rutin)  Quercetin-3-O-β-glucuronide  Quercetin-3-O-β-D-galactoside (hyperoside)  Quercetin-3-O-β-D- glucoside (isoquercitrin)* | Flavonoids | Zekic et al. 2021  Papp et al. 2004  Apati et al. 2002 |
| 12.5 | Quercetin pentoside | Flavonoids | Zekic et al. 2021 |
| 13.2,  13.3 | Quercetin,  Kaempferol | Flavonoids  phenolic acids | Papp et al. 2004  Apati et al. 2002 |
| 16.5 | Quercetin-3-O-β-D-rhamnoside (quercitrin)*,  Hyperoside (quercetin-3-O-galactoside) | Flavonoids | Apati et al. 2002  Judžentienė et al 2023 |
| 17.2, 17.6 | Kaempferol-(acetyl)-hexoside,  Isorhamnetin-(acetyl)-hexoside | Flavonoids | Zekic et al. 2021 |
| 19.0, 19.7 | Kampferol-3-O-α-L-rhamnoside (afzelin), Nicotiflorin | Flavonoids | Apati et al. 2002  Papp et al. 2004 |
| 20.4 | Rutin | Flavonoid | Papp et al. 2004 |
| 21.2, 21.4 | Hyperoside, isoquercitrin | Flavonoids | Papp et al. 2004 |
| 22.6 | Cyanidin 3-O-glucosyl rutinoside | Flavonoid | Judžentienė et al 2023 |
| 24.2  24.5 | Kaemperfol glycoside  Quercitrin | Flavonoids | Judžentienė et al 2023  Papp et al. 2004 |
| 26.5 | Afzelin | Flavonoid | Papp et al. 2004 |
| 28.0, 28.8, 30.2 | Dicaffeoylquinic acid | Phenolic acid | Wu et al. 2022 |
| 35.4 | Cinnamtannin A2 | Condensed tannin | Judžentienė et al 2023 |
| 36.1, 38.9, 44.5 | Caffeoylquinic acid | phenolic acid | Gobbo-Neto et al. 2008 |
| 45.3, 46.1, 48.7, 49.6, 53.8, 55.7 | Caffeoylferuloylquinic acid | phenolic acid | Gobbo-Neto et al. 2008 |
| 50.9 | 15-hydroxyeremantholide C | Sesquiterpene lactone | Gobbo-Neto et al. 2008 |
| 52.5 | Casticin | Flavonoid | Wu et al. 2022 |
| 55.0 | 3,4-di-O-(E)-feruloylquinic acid | phenolic acid | Gobbo-Neto et al. 2008 |
| 58.3 | Pinobanksin | Flavonoid | Gobbo-Neto et al. 2008 |

Table S5a Measurement model quality: composite reliability (CR), average variance extracted (AVE), and indicator loadings for each latent variable in the PLS-PM analysis.

| Latent Variable | Type | Items | Composite Reliability | AVE | Loading Range |
| --- | --- | --- | --- | --- | --- |
| Invasion community | Exogenous | 2 | 0.78 | 0.64 | 0.790-0.812 |
| AMF community | Endogenous | 3 | 0.96 | 0.90 | 0.890-0.992 |
| Soil abiotic property | Endogenous | 3 | Mode B | Mode B | -0.916-0.863 |
| Leaf chemistry | Endogenous | 2 | 0.97 | 0.95 | 0.970-0.976 |
| Root chemistry | Endogenous | 3 | 0.80 | 0.58 | 0.494-0.932 |
| Allelopathic potential | Endogenous | 6 | Mode B | Mode B | -0.156-0.511 |

Table S5b Structural model results: coefficients of determination (*R²*) for all endogenous latent variables.

| Endogenous Variable | *R²* | Interpretation |
| --- | --- | --- |
| AMF community | 0.279 | Moderate |
| Soil abiotic property | 0.443 | Moderate |
| Leaf chemistry | 0.366 | Moderate |
| Root chemistry | 0.447 | Moderate |
| Allelopathic potential | 0.677 | Strong |

Table S5c Direct effects with bootstrap validation: standardized path coefficients and their 95% confidence intervals for key relationships in the PLS-PM model.

| Path | Type | Coefficient | 95% CI | *P*-value |
| --- | --- | --- | --- | --- |
| Invasion community → AMF community | Direct | -0.529 | [-0.830, 0.697] | 0.024 |
| Invasion community → Soil abiotic property | Direct | 0.615 | [-0.722, 1.000] | 0.016 |
| AMF community → Soil abiotic property | Direct | 0.738 | [-0.085, 1.067] | 0.005 |
| AMF community → Leaf chemistry | Direct | -0.529 | [-0.920, -0.070] | 0.033 |
| AMF community → Root chemistry | Direct | 0.332 | [-0.420, 0.683] | 0.136 |
| Soil abiotic property → Leaf chemistry | Direct | -0.148 | [-0.574, 0.655] | 0.521 |
| Soil abiotic property → Root chemistry | Direct | -0.733 | [-1.111, 0.847] | 0.003 |
| Leaf chemistry → Allelopathic potential | Direct | 0.471 | [-0.794, 0.777] | 0.006 |
| Root chemistry → Allelopathic potential | Direct | 0.698 | [-0.821, 0.943] | < 0.001 |


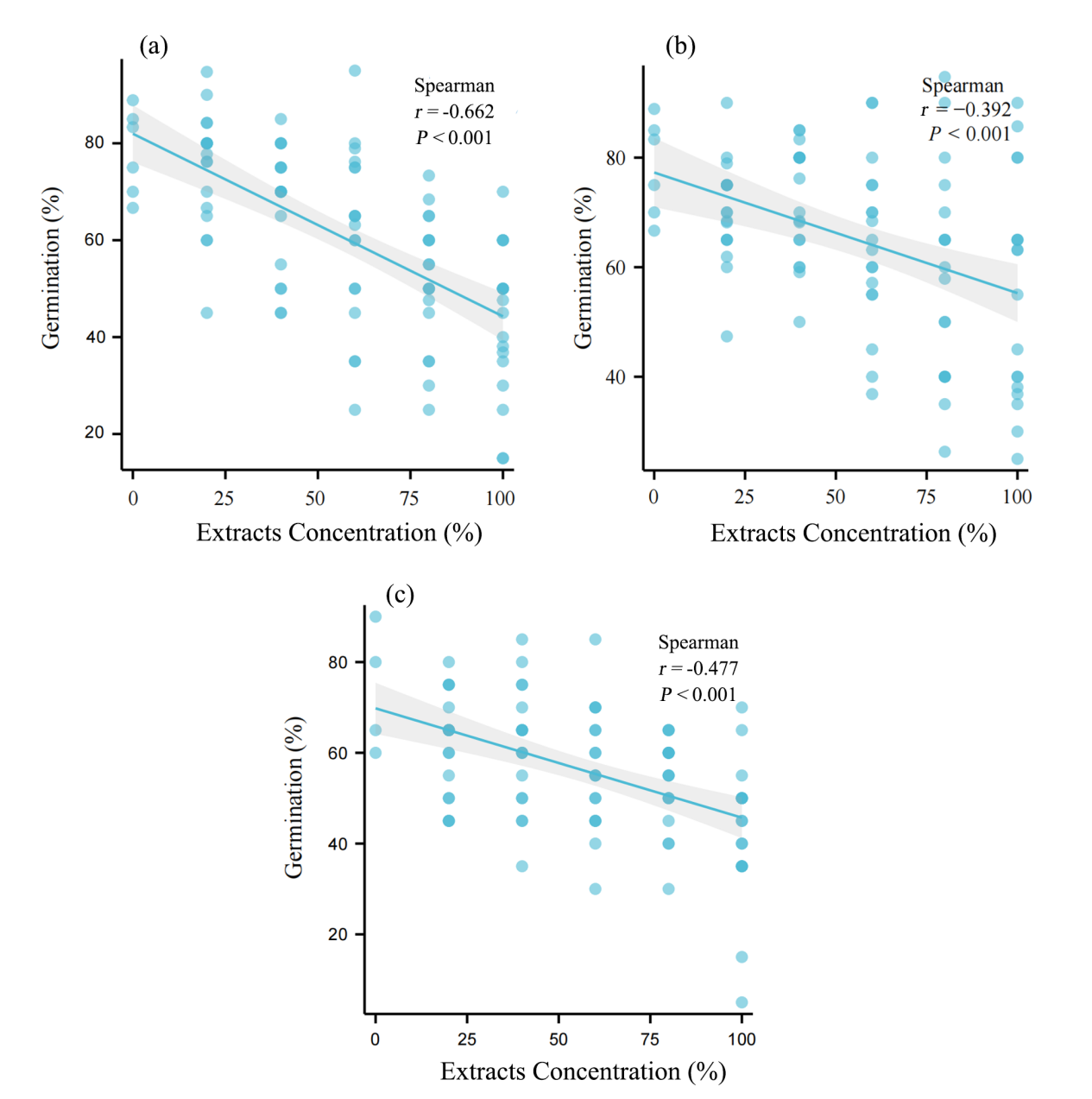


Fig. S1 Correlation between leaf **(a) and** root **(b)** extract concentrations of S. canadensis and seed germination of R. sativus

**
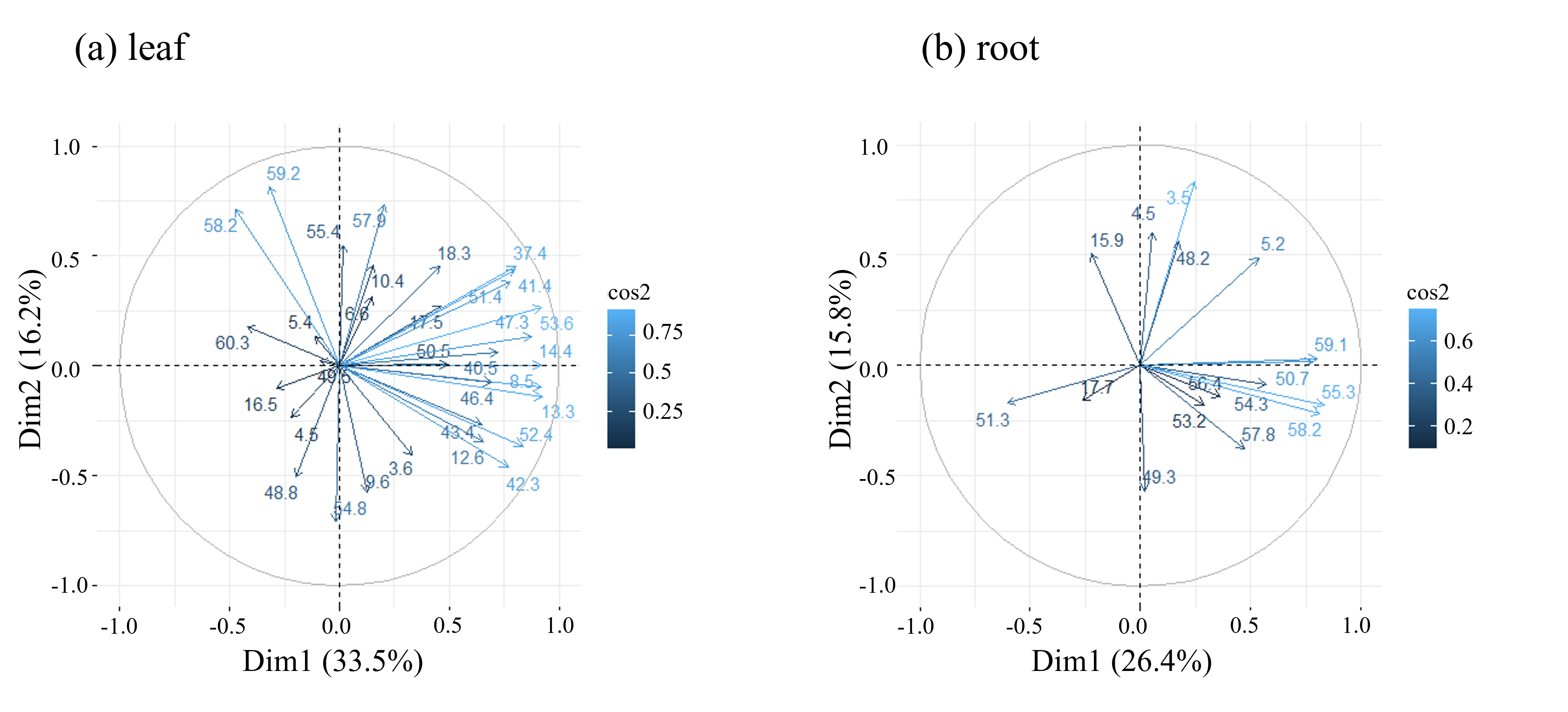
**

Fig. S2 Principal component analysis (PCA) of chemical compounds identified in leaf (a) and root (b) extracts (numbers indicate retention times).


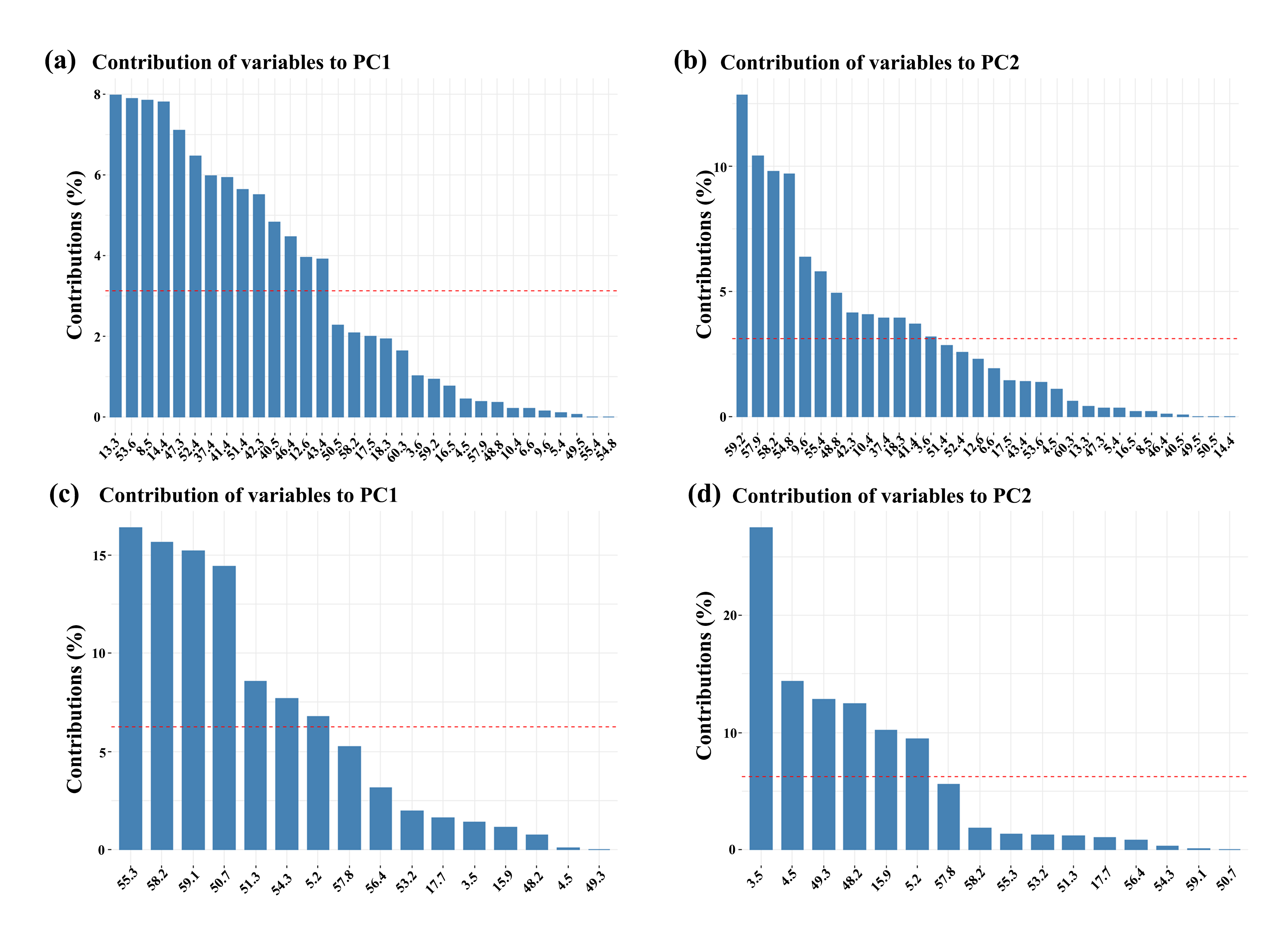
Fig. S3 The contributions of feature peaks in leaf extracts (a, b) and in root extracts (c, d) to the first four principal components. The eigenvectors with values higher than the red dashed lines, corresponding to the average contributions of each trait to the corresponding PC, are the traits that are most strongly correlated with the corresponding PC axis. The numbers in the first axis indicate the chromatographic peaks at different retention time.


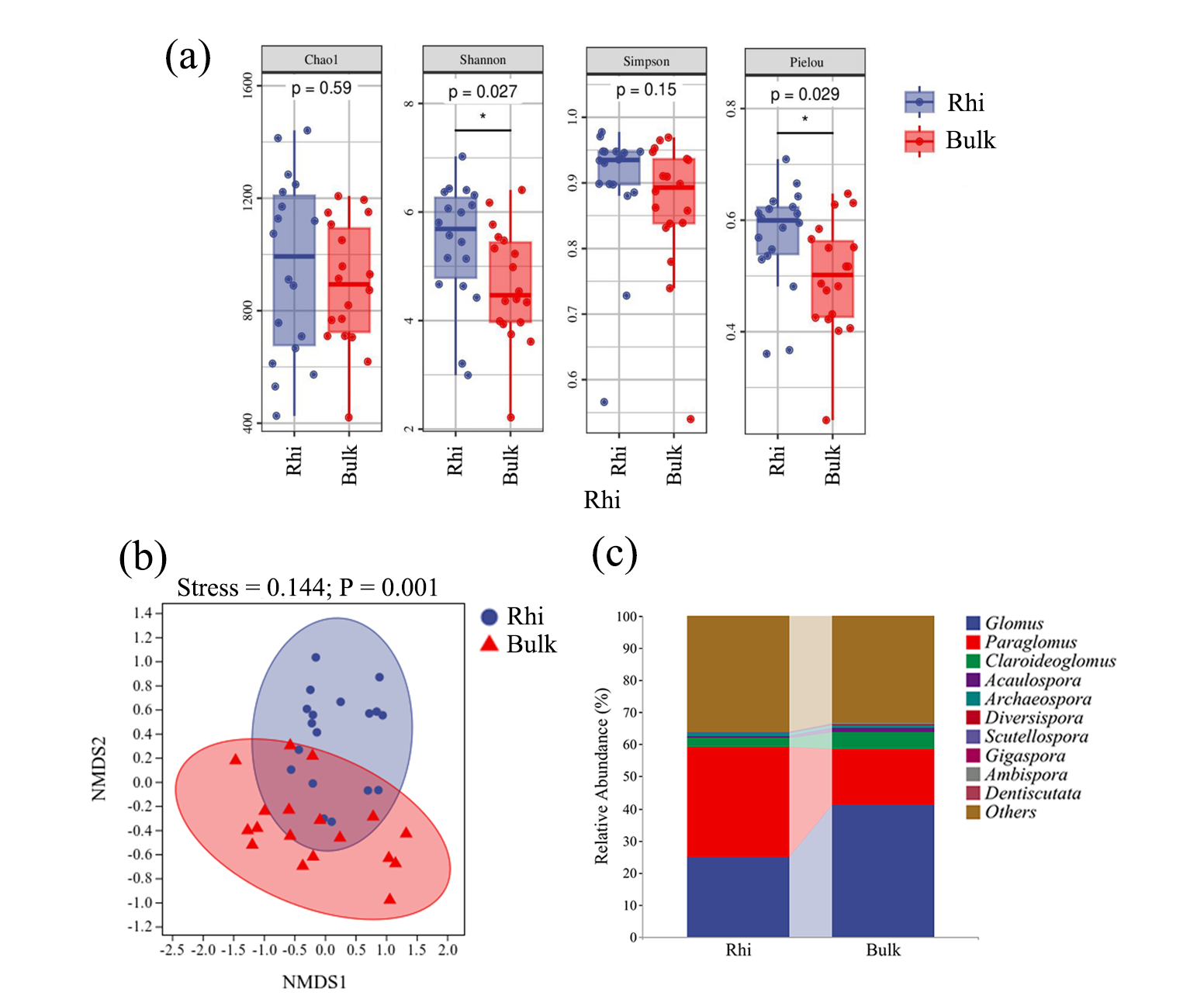


Fig. S4 Alpha diversity indices (a), Nonmetric multidimensional scaling results (NMDS) (b) and (c) The relative abundance of AMF communities in the bulk and rhizosphere soils of S. canadensis. Rhi and bulk indicate rhizosphere and bulk AMF communities.

**References**

Apati P, Szentmihalyi K, Balazs A, Baurnann D, Harnburge M, Krist TS, Szoke E (2002) HPLC analysis of the flavonoids in pharmaceutical preparations from canadian goldenrod (*Solidago canadensis*). *Chromatographia* **56:** 65–68.

Judžentienė A, Būdienė J, Labanauskas L, Stancelytė D, Nedveckytė I (2023) Allelopathic activity of canadian goldenrod (*solidago canadensis* l.) extracts on seed germination and growth of lettuce (*lactuca sativa* l.) and garden pepper cress (*lepidium sativum* l.) *Plants* **12:** 1421.

Papp I, Apati P, Andrasek V, Blazovics A, Balazs A, Kursinszki L, Kite GC, Houghton, PJ, Kery A (2004) LC-MS analysis of antioxidant plant phenoloids. *Chromatographia* **60:** 93-100.

Wu TF, Ma ZG, Zhang Y, Wu MH, Cao H (2022) Simultaneous quantitative analysis of 11 constituents in Viticis Fructus by HPLC-HRMS and HPLC-DAD combined with chemometric methods. *Phytochemical Analysis* **34:** 163-174.

Gobbo-Neto L, Lopes NP (2008) Online identification of chlorogenic acids, sesquiterpene lactones, and flavonoids in the brazilian arnica *Lychnophora ericoides* Mart. (Asteraceae) leaves by HPLC-DAD-MS and HPLC-DAD-MS/MS and a validated HPLC-DAD method for their simultaneous analysis. *Journal of Agricultural and Food Chemistry* **56:** 1193-1240.

Zekic J, Vovk I, Glavnik V (2021) Extraction and analyses of flavonoids and phenolic acids from canadian goldenrod and giant goldenrod. *Forests* **12:** 1.
